# Oregano Oil Combined with Macleaya Cordata Oral Solution Improves the Growth Performance and Immune Response of Broilers

**DOI:** 10.3390/ani12182480

**Published:** 2022-09-19

**Authors:** Cheng Zhang, Weihao Li, Ligong Chen, Zhaoliang Chen, Xuejing Wang, Qianqian Xu, Hailong Zhang, Huan Chen, Juxiang Liu

**Affiliations:** College of Veterinary Medicine, Hebei Agricultural University, Baoding 071000, China

**Keywords:** oregano oil, macleaya cordata, oral solution, broilers, growth performance

## Abstract

**Simple Summary:**

Developing safe and effective antibiotic growth promoters (AGPs) substitutes is particularly important to improve animal health and production performance. As an essential plant oil, the oregano oil's main bioactive substance is carvacrol, which has been proven to have antioxidant, anti-inflammatory, antibacterial, and antiviral properties. The sanguinarine from macleaya cordata is the primary bioactive substance. Sanguinarine has anti-tumor, immune-enhancing, antibacterial, and anti-inflammatory effects. However, it has not been reported whether the compatibility of oregano oil and macleaya cordata extract could produce better results. This study is the first to report the effect of oregano oil combined with macleaya cordata oral solution on the growth of broilers. The oregano oil combined with macleaya cordata oral solution significantly improved the growth performance of broilers. At the same time, serum biochemical indices, serum antioxidant indices, serum immune indices, serum cytokines, and intestinal morphology were significantly improved. In summary, our results demonstrated that the mixed solution of oregano oil and macleaya cordata has substantial potential to be an alternative to AGPs for broilers to reduce costs and improve benefits. This study provides basic data and technical support for further research.

**Abstract:**

The abuse of AGPs in animal husbandry has led to severe problems such as drug resistance and ecological, and environmental destruction, which seriously threaten human health and public health security. In recent years, extracts of oregano oil and macleaya cordata have become a hot spot in the research and application of AGP substitutes for their safety and high efficiency. This study is the first to report the effect of oregano oil combined with macleaya cordata oral solution on broiler growth performance. A total of 960 one-day-old broiler chickens were randomly divided into four treatment groups (240 chickens per group). Each treatment group was divided into six replicate groups (40 birds per replicate group). There were four groups in this study: the solvent control group, the oregano essential oil combined with macleaya cordata extract oral solution group (OS group), the oregano essential oil oral solution group (OEO group), and the macleaya cordata extract oral solution group (MCE group). Two chickens from each replicate group were collected and mixed into a composite sample. Six composite samples were obtained for each treatment group. The results showed that the oregano oil combined with macleaya cordata oral solution significantly improved the growth performance of broiler chickens. At the same time, serum biochemical indices, serum antioxidant indices, serum immune indices, serum cytokines, and intestinal morphology were significantly improved by the OS group. This study shows that oregano oil combined with macleaya cordata oral solution has substantial potential to be an alternative to AGPs for broilers.

## 1. Introduction

With the development of animal husbandry, intensive breeding technology has been promoted, and the breeding density of broiler chickens has gradually increased. In intensive farming systems, broilers are often exposed to various adverse factors, such as inadequate nutrition, poor environment, and disease, resulting in impaired growth, poor health, and even high mortality [1]. Additionally, pathogenic bacteria on the farm could cause diseases in broilers, leading to a large number of poultry deaths and huge economic losses [2]. To improve productivity and prevent disease, antibiotic growth promoters (AGPs) have been widely used as performance-enhancing feed additives in poultry production [3]. The widespread use of AGPs, however, does cause not only antibiotic resistance, but also antibiotic residues in animal products, and residual antibiotics contaminate the environment through feces, posing a great threat to public health security [4,5]. China, the largest consumer of veterinary antibiotics, has banned the use of AGPs in animal feed since 2020, which may reduce the growth performance of animals and their ability to resist disease [6]. Therefore, developing safe and effective AGPs substitutes is particularly important to improve animal health and production performance.

Oregano oil is an essential oil derived from oregano, a fragrant plant widely grown in China and used primarily as an herb [7]. As an essential plant oil, the oregano oil’s main bioactive substance is carvacrol, which has been proven to have antioxidant, anti-inflammatory, antibacterial, and antiviral properties in previous studies [8]. A growing number of studies have shown that diets supplemented with oregano oil can promote performance and improve the antioxidant status and immunity of broilers [9,10]. Macleaya cordata is a perennial herb of the poppy family, widely distributed in southern China [11]. Previous studies have also shown that sanguinarine from macleaya cordata is the main bioactive substance [12]. Sanguinarine has an anti-tumor, immune-enhancing [13], antibacterial [14], and anti-inflammatory effects [15]. Dietary supplementation with macleaya cordata extracts improves animal performance, immunity, and intestinal health [16]. However, it has not been reported whether the compatibility of oregano oil and macleaya cordata extract will produce better results. Meanwhile, oregano oil and macleaya cordata extract were applied in the form of feed premix which can only be used by adding them to the feed. There are significant problems such as uneven mixing and increasing the workload of feeders. Moreover, premix cannot be used in water and cannot adapt to the waterline system of modern commercial farming of broilers.

In this study, oregano oil and macleaya extract were prepared in oral liquid dosage form, which improved some practical production problems. For the first time, the oregano oil combined with macleaya cordata oral solution was investigated to improve growth performance and immune response in broilers. The potential of the combination of the oregano oil combined with macleaya cordata oral solution as an alternative to AGPs in broilers was also demonstrated.

## 2. Materials and Methods

### 2.1. Reagents

Oregano oil (the carvacrol content was 45 mg/mL) (Cat.NO: O137777) and 2,6-di-tert-butyl-4-methylphenol (Cat.NO: D104363) were purchased from Aladdin Biochemical Technology Co. Ltd. (Aladdin, Shanghai, China). Macleaya cordata extract (the sanguinarine content was 6.5 mg /mL) (Cat.NO: MC70) was purchased from Hanqing Biotechnology Co., Ltd. (Hunan, China). Polyoxyl-40 hydrogenated castor oil (Cat.NO: 07076) was purchased from Sigma (Sigma Chemical Company, St Louis, MO, USA).

### 2.2. Oral Solution Preparation

Four different oral solutions were used in this study. The oral solution was configured according to our previous study [17]. The first oral liquid was excipient solvent (solvent control) (25% solubilizing agent, polyoxyl-40 hydrogenated castor oil, and 0.02% 2,6-di-tert-butyl-4-methylphenol, the rest was sterile water). The second was oregano oil and macleaya cordata extract mixed oral solution (OS: 5% oregano oil, 1% macleaya cordata extract, 25% solubilizing agent, polyoxyl-40 hydrogenated castor oil, and 0.02% 2,6-di-tert-butyl-4-methylphenol, the rest was sterile water). The third was oregano oil oral solution (OEO: 5% oregano oil, 25% solubilizing agent, polyoxyl-40 hydrogenated castor oil, and 0.02% 2,6-di-tert-butyl-4-methylphenol, the rest was sterile water). The fourth was macleaya cordata oral solution (MDE: 1% macleaya cordata extract, 25% solubilizing agent, polyoxyl-40 hydrogenated castor oil, and 0.02% 2,6-di-tert-butyl-4-methylphenol, the rest was sterile water).

### 2.3. Animal Experiments and Sample Collection

#### 2.3.1. Birds, Housing, and Experimental Groups

**Figure 1:**
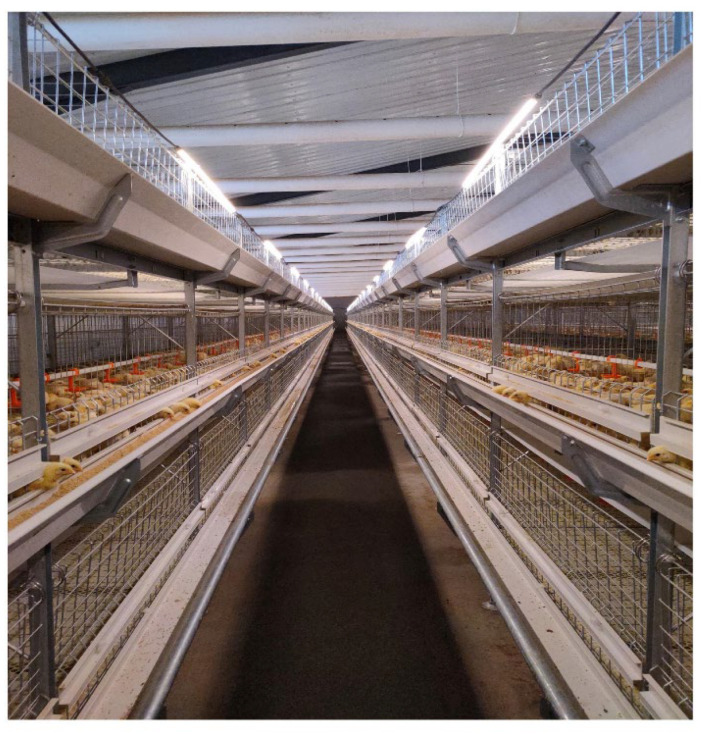
Structure of broiler house and waterline system.

A total of 960 one-day-old, white-feathered broilers (Hubbard broiler) were purchased from Enkang Animal Husbandry Co., Ltd. (Hebei, China) and randomly divided into 4 treatment groups (*n* = 240/treatment). Each treatment group had 6 replicates consisting of 40 broilers in each replicate. Three cages were used in the chicken houses of white-feathered broilers (Figure 1), with good ventilation and lighting. The indoor temperature was maintained at 22–25 °C, and the humidity was maintained at 50–60%. The ration was fed automatically and regularly (6:00 in the morning and 3:00 in the afternoon). The diet formula is shown in Table 1. Diets were formulated following the nutrient requirement recommendations of the National Research Council (NRC, 1994) [18]. An automated waterline system was used in the broiler house (Figure 1). All chickens received feed and water ad libitum. The four treatment groups were the solvent control group (control group), oregano oil and macleaya cordata mixed oral solution group (OS group), oregano oil oral solution group (OEO group), and macleaya cordata oral solution group (MCE group). The four oral solutions were added to the waterline system of the corresponding four treatment groups (control group, OS group, OEO group, and MCE group) every day. The dosage of oral liquid was 125 mL in 1000 L water.

#### 2.3.2. Sample Collection

At the end of the study (day 42), all chickens fasted for 12 h. The chickens were euthanized by cervical dislocation, and their blood was collected into sterile tubes. Blood samples were allowed to clot at room temperature, centrifuged at 4000× *g* for 15 min at 4 °C, and serum supernatants were collected after centrifugation. Based on a previous study, our protocol for collecting samples and testing was to randomly collect 48 chickens from the 4 treatment groups (12 chickens per group), 2 chickens from each replicate, and samples from both chickens mixed for testing [19]. All serum samples were stored at −80 °C until further analysis. For histological examination, various parts of the small intestine (duodenum, jejunum, ileum) were removed and fixed in a 10% formalin solution.

### 2.4. Data Recording and Experimental Testing

According to previous studies, broilers were weighed at the age of 1 day. During the experiment, the growth conditions and mortality of the broilers were observed every day. The amount of feed offered and refused by each group was recorded to calculate the average daily feed intake (ADFI), average daily gain (ADG), and average daily feed to gain ratio (F/G) [20]. The following kits used for the determination of serum biochemical indices were purchased from the Jiancheng institute of biological engineering (Nanjing, China): total protein (TP) quantitative assay kit (A045-2-2), albumin (ALB) assay kit (A028-1-1), urea (BUN) assay kit (C013-2-1), triglyceride (TG) assay kit (A110-2-1), total cholesterol (TC) assay kit (A111-2-1), and alkaline phosphatase (ALP) assay kit (A059-2-1). The following kits used to determine the serum antioxidant index were purchased from Jiancheng institute of biological engineering (Nanjing, China): micro total antioxidant capacity (T-AOC) assay kit (A015-1-1), glutathione peroxidase (GSH-PX) assay kit (A005-1-2), malondialdehyde (MDA) assay kit (A003-1-2), and superoxide dismutase (SOD) assay kit (A001-3-2). The following kits were used to determine serum immune indices: chicken IgA (Immunoglobulin A) ELISA kit and chicken IgM (immunoglobulin M) ELISA kit purchased from Abcam (Cambridge, UK) and chicken IgG (immunoglobulin G) ELISA kit (DL-IGG-CH) purchased from Donglin Technology Co., LTD. (Wuxi, China). The following kits used for determining serum cytokines were purchased from Fien Biotechnology Co., Ltd. (Nanjing, China): chicken IFN-α ELISA kit (ECH0024) ELISA kit (ECH0040), chicken IL-4 (interleukin 4) ELISA kit (ECH0044), and chicken IL-6 (interleukin 6) ELISA kit (ECH0046). All assay procedures were performed in strict accordance with the kit instructions. Image-Pro software (Media Cybernetics, Rockville, MD, USA) was applied to calculate villus height, crypt depth, and villus height/crypt depth according to previously reported methods [21].

### 2.5. Statistical Analysis

Means and standard deviations (SD) are the results. Statistical differences were assessed by one-way analysis of variance using GraphPad Prism 8 software (GraphPad Prism Software, San Diego, CA, USA) and the significance of the differences was estimated by Tukey’s honestly significant difference test (Tukey HSD). *p* < 0.05 was considered statistically significant.

## 3. Results

### 3.1. Evaluation of In Vivo Growth Promotion

**Figure 2:**
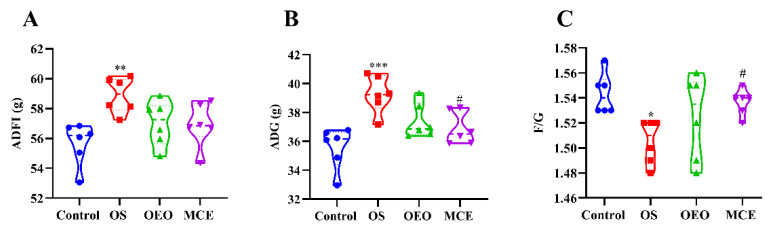
Growth performance of broilers. (**A**) Average daily feed intake (ADFI). (**B**) Average daily gain (ADG). (**C**) Average daily feed to gain ratio (F/G). * *p* < 0.05, ** *p* < 0.01, *** *p* < 0.001, vs. control group; ^#^ *p* < 0.05, vs. OS group (*n* = 6).

The effects of different treatment groups on the growth performance of broilers are shown in Figure 2 and Table S1. All chickens remained healthy, and no mortality was observed throughout the whole experimental period. The mean of ADFI (58.90 ± 1.19 g) and ADG (39.25 ± 1.29 g) for the OS group was significantly greater than that for the control group (55.68 ± 1.44 g and 35.59 ± 1.45 g) (*p* < 0.01). The OS group (1.51 ± 0.02) was significantly lower than the mean of the MCE group (56.93 ± 1.48) for ADFI/ADG (*p* < 0.05). The ADFI (57.02 ± 1.51 g) and ADG (37.39 ± 1.21 g) of the OEO group and MCE group (56.93 ± 1.48 g and 36.90 ± 1.13 g) also showed an increasing trend but did not show any significant differences (*p* > 0.05). ADG (36.90 ± 1.13) and ADFI/ADG (1.54 ± 0.01) in the MCE group were significantly different compared with the OS group (39.25 ± 1.29 and 1.51 ± 0.02) (*p* < 0.05).

### 3.2. Serum Biochemical Indices

**Figure 3:**
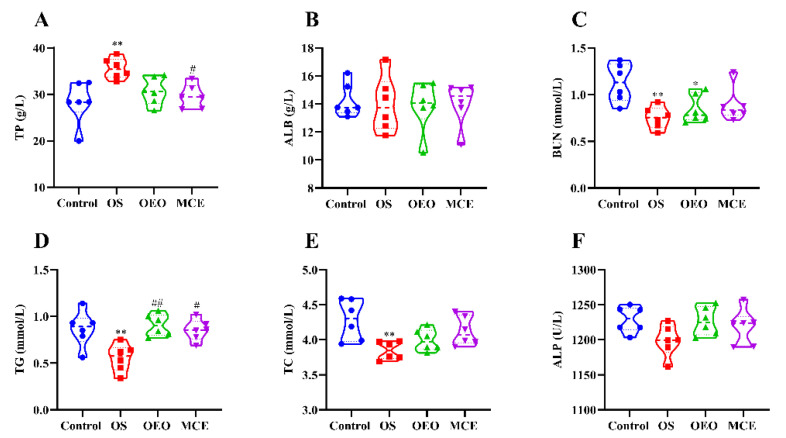
Serum biochemical parameters of broilers. (**A**) Total protein (TP). (**B**) Albumin (ALB). (**C**) Urea nitrogen (BUN). (**D**) Triglycerides (TG). (**E**) Total cholesterol (TC). (**F**) Alkaline phosphatase (ALP). * *p* < 0.05, ** *p* < 0.01, vs. control group; ^#^ *p* < 0.05, ^##^ *p* < 0.01, vs. OS group (*n* = 6).

Figure 3 and Table S1 show the changes in serum biochemical parameters of broilers in different treatment groups. The total protein level for the OS group was 35.59 ± 2.18 g/L, which was significantly greater than that for the control group (28.32 ± 4.56 g/L) (*p* < 0.01). Serum urea levels in OS (0.75 ± 0.12 mmol/L) and OEO (0.85 ± 0.15 mmol/L) groups were significantly lower than that of the control group (1.13 ± 0.21 mmol/L) (*p* < 0.01 and *p* < 0.05). Serum triglyceride (0.56 ± 0.15 mmol/L) and total cholesterol (3.85 ± 0.13 mmol/L) levels were significantly lower in the OS group than that of the control group (0.87 ± 0.19 mmol/L and 4.29 ± 0.29 mmol/L) (*p* < 0.01). The total protein levels of the MCE group (29.55 ± 2.56 g/L) were statistically significantly lower than the OS group (35.59 ± 2.18 g/L) (*p* < 0.05).

### 3.3. Serum Antioxidant Indices

**Figure 4:**
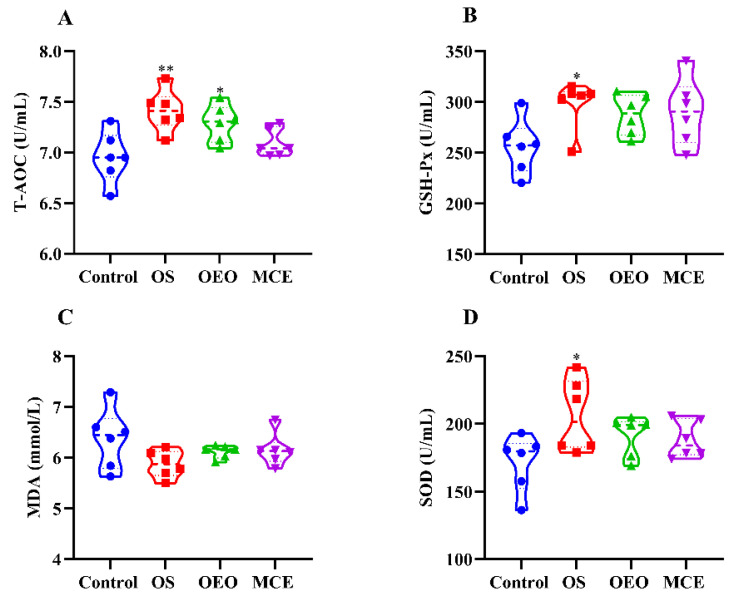
Serum antioxidant indices of broilers. (**A**) Total antioxidant capacity (T-AOC). (**B**) Glutathione peroxidase (GSH-PX). (**C**) Malondialdehyde (MDA). (**D**) Superoxide dismutase (SOD). * *p* < 0.05, ** *p* < 0.01, vs. control group (*n* = 6).

Figure 4 and Table S1 show the effect of different treatment groups on the serum antioxidant index of broilers. Compared with the control group (6.95 ± 0.25 U/mL), the total antioxidant capacity values of the OS group (7.41 ± 0.21 U/mL) and OEO group (7.29 ± 0.18 U/mL) were significantly increased (*p* < 0.05). Serum concentrations of glutathione peroxidase (298.59 ± 23.69 U/mL) and superoxide dismutase (205.89 ± 26.87 U/mL) were significantly in the OS group than that in the control group (255.96 ± 26.87 U/mL and 171.57 ± 20.89 U/mL) (*p* < 0.05).

### 3.4. Serum Immune Indices

**Figure 5:**
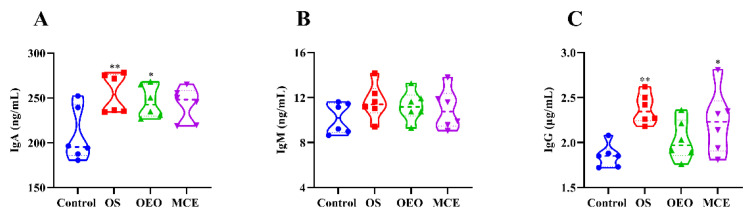
Serum immune indices of broilers. (**A**) Immunoglobulin A (IgA). (**B**) Immunoglobulin M (IgM). (**C**) Immunoglobulin G (IgG). * *p* < 0.05, ** *p* < 0.01, vs. control group (*n* = 6).

Figure 5 and Table S1 show the changes in serum immunological indexes of broiler chickens in different treatment groups. Serum immunoglobulin A (IgA) levels were significantly increased in the OS (255.13 ± 21.87 U/mL) and OEO groups (245.87 ± 17.89 U/mL) compared to the control group (208.33 ± 29.87 U/mL) (*p* < 0.05). Serum immunoglobulin G (IgG) levels were significantly higher in the OS (2.38 ± 0.17 ng/mL) and MCE groups (2.23 ± 0.35 ng/mL) than that in the control group (1.85 ± 0.13 ng/mL) (*p* < 0.05).

### 3.5. Serum Inflammatory Cytokines

**Figure 6:**
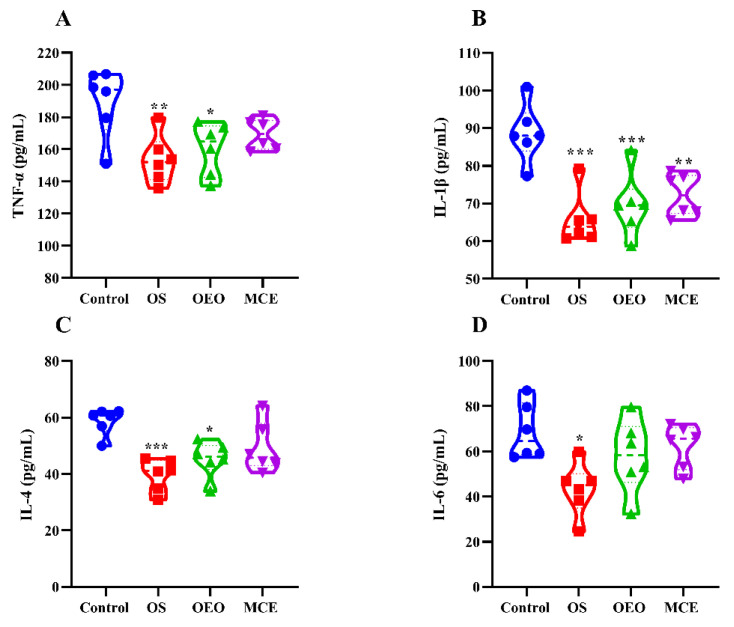
Serum cytokines of broilers. (**A**) Tumor necrosis factor-α (TNF-α). (**B**) Interleukin 1β (IL-1β). (**C**) Interleukin 4 (IL-4). (**D**) Interleukin 6 (IL-6). * *p* < 0.05, ** *p* < 0.01, *** *p* < 0.001, vs. control group (*n* = 6).

Figure 6 and Table S1 show the effect of cytokines on the serum cytokines of broilers in different treatment groups. Compared with the control group, the levels of tumor necrosis factor-α (TNF-α) and interleukin-1β (IL-1β) in the serum of the broilers in the OS group (153.68 ± 15.26 pg/mL and 65.78 ± 6.92 pg/mL) and OEO group (160.26 ± 16.32 pg/mL and 69.56 ± 8.35 pg/mL) were significantly lower than that in the control group (189.56 ± 21.34 pg/mL and 88.66 ± 7.73 pg/mL) (*p* < 0.05). The levels of interleukin 4 (IL-4) in the OS group (39.67 ± 5.72 pg/mL) and OEO group (45.29 ± 6.31 pg/mL) were significantly lower than that in the control group (58.74 ± 4.69 pg/mL) (*p* < 0.05), and the level of interleukin 6 (IL-6) in the OS group (43.26 ± 11.59 pg/mL) was significantly lower than that in the control group (68.65 ± 12.34 pg/mL) (*p* < 0.05).

### 3.6. Changes in the Intestinal Morphology of Broilers

**Figure 7:**
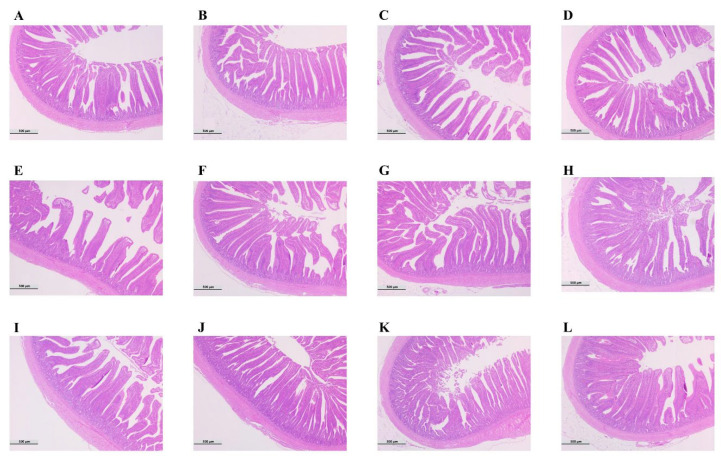
Intestinal wall morphology in broilers. (**A**) Duodenum of the control group. (**B**) Duodenum of the OS group. (**C**) Duodenum of the OEO group. (**D**) Duodenum of the MCE group. (**E**) Jejunum of the control group. (**F**) Jejunum of the OS group. (**G**) Jejunum of the OEO group. (**H**) Jejunum of the MCE group. (**I**) Ileum of the control group. (**J**) Ileum of the OS group. (**K**) Ileum of the OEO group. (**L**) Ileum of the MCE group. Images were obtained at 40× magnification.

**Figure 8:**
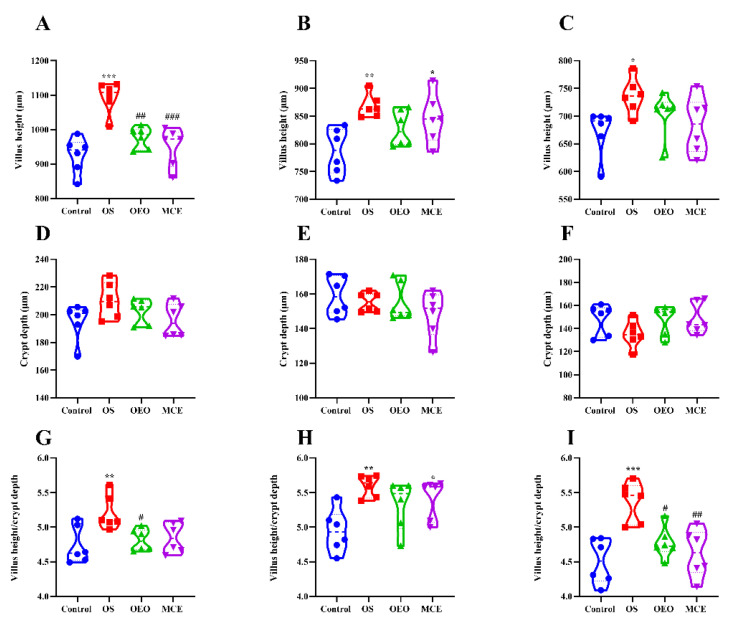
The villus height, crypt depth, and villus height/crypt depth ratio of broilers. (**A**) Villus height of the duodenum. (**B**) Villus height of the jejunum. (**C**) Villus height of the ileum. (**D**) Crypt depth of the duodenum. (**E**) Crypt depth of the jejunum. (**F**) Crypt depth of the ileum. (**G**) Villus height/crypt depth ratio in the duodenum. (**H**) Villus height/crypt depth in the jejunum. (**I**) Villus height/crypt depth in the ileum. * *p* < 0.05, ** *p* < 0.01, *** *p* < 0.001, vs. control group; ^#^ *p* < 0.05, ^##^ *p* < 0.01, ^###^ *p* < 0.001, vs. OS group (*n* = 6).

Figure 7 and Figure 8 show changes in broiler gut morphology, villus height, crypt depth, and villus height/crypt depth for different treatment groups. Histological examination showed that the OS group had the best intestinal structure. The results showed a very significant increase in villus height and villus height/crypt depth of the duodenum in the OS group (1094.27 ± 46.17 and 5.21 ± 0.25) compared with the control group (925.73 ± 51.99 and 4.74 ± 0.27) (*p* < 0.01), and the villus height of which was significantly greater than that for the OEO group (976.31 ± 31.15 μm) and the MCE group (950.32 ± 56.08 μm) (*p* < 0.01). Moreover, the villus height/crypt depth in the OEO group (4.82 ± 0.16) was significantly lower than that in the OS group (*p* < 0.05). In the jejunum, villus height and villus height/crypt depth were significantly increased in the OS (867.91 ± 21.08 μm and 5.21 ± 0.25) and MCE groups (845.53 ± 44.73 μm and 4.85 ± 0.22) compared with the control group (786.61 ± 41.12 μm and 4.74 ± 0.27) (*p* < 0.05). Compared with the control group (672.57 ± 42.35 μm and 4.51 ± 0.33), ileal villus height and villus height/crypt depth were significantly increased in the OS group (736.57 ± 32.11 μm and 5.37 ± 0.29) (*p* < 0.05). Compared with the OS group, the villus height/crypt depth between the OEO group (4.78 ± 0.23) and the MCE group (4.62 ± 0.35) was statistically lower (*p* < 0.05).

## 4. Discussion

In this study, extracts of oregano oil and macleaya cordata were prepared into an oral liquid for the first time. Using an oral liquid form can greatly reduce the feeding load and cost. Previous studies only added oregano oil or macleaya cordata extracts into the fodder, but uneven distribution and rapid change in additive properties cannot effectively improve the curative effects [15,19]. Therefore, adding oral liquid directly to the waterline system of the chicken house has an excellent regulatory impact on the growth performance of broiler chickens.

In recent years, macleaya cordata has been added to the diets of pigs, cattle, chickens, and fish. The addition of 20 mg/kg macleaya cordata extracts significantly increased the performance of broilers. Dietary supplementation with oregano oil has also been reported to significantly improve the growth capacity of broilers [20,22,23,24]. This is consistent with the results of our study, which showed that oregano oil combined with macleaya cordata oral solution has a more significant effect on improving the growth ability of broilers. The serum biochemical index is an important index reflecting the metabolic change and tissue function of the body. Serum protein content reflects the digestion and absorption of protein and body immunity, while serum urea nitrogen content reflects protein metabolism and amino acid balance in animals [22]. Serum triglyceride and cholesterol contents reflect the lipid metabolism status of animals [25]. Excessive triglyceride content in serum will lead to metabolic diseases in the body, and oregano oil combined with macleaya cordata oral solution could reduce the possibility of metabolic diseases. Serum alkaline phosphatase to some extent can reflect the damage level of the cells under normal circumstances and low serum alkaline phosphatase activity. When cells are subjected to various factors, such as stress stimulation and damage, cell membrane permeability increases, alkaline phosphatase is released into the blood speed increase and there is a significant rise in serum ALP activity [25]. In this study, the oregano oil combined with macleaya cordata oral solution significantly improved serum biochemical indices, which is consistent with the effect of the extracts of oregano oil or macleaya cordata previously reported [23,26].

Free radicals in the process of nutrient metabolism are produced in body metabolism, and the excess free radicals can cause liposome reactions, resulting in cell or tissue damage, and then causing a series of diseases. The animal body contains SOD, GSH-PX, and other enzyme systems, which can be induced to synthesize due to the increased number of free radical compounds in the body. The increased enzymatic activity of this system indicates that the protection of cells from free radical damage is enhanced [27]. T-AOC is a comprehensive index used to measure the antioxidant capacity of the body [28]. As the final product of lipid peroxidation, MDA content can directly reflect the degree of cell membrane oxidation [29]. The results showed that the oregano oil combined with macleaya cordata oral solution could significantly improve the total antioxidant capacity of broilers, significantly increase the activities of superoxide dismutase and glutathione peroxidase in the serum of broilers and reduce the content of malondialdehyde in the serum of broilers to a certain extent.

Immunoglobulin in serum is a globulin that has antibody activity and the immune response of the broiler directly. Immunoglobulin content is of great significance in revealing the immune response ability of broilers. Detection of IgA, IgG, and IgM contents in serum can represent the level of immunoglobulin in serum [30,31]. Studies have shown that the addition of oregano oil or macleaya cordata extracts can improve serum immune indices [19,32]. Our results are consistent with previous reports that the oregano oil combined with macleaya cordata oral solution can significantly increase the contents of IgA and IgG in the serum of broilers. However, there was no significant difference in IgM, which may be caused by our dosage form and content.

The content of inflammatory factors in serum was also determined. TNF-α, a pro-inflammatory cytokine with diverse physiological functions, has been reported to induce apoptosis in intestinal epithelial cells [33]. IL-6 is involved in the development of inflammation by increasing other inflammatory cytokines [34]. Prior research indicates that colon mucosal macrophages increase in patients with inflammatory diseases, as well as the expression of the inflammatory factors TNF-α and IL-6 [35]. IL-1β and IL-4 play an important role in the inflammatory response of broilers [36]. Previous studies have shown that macleaya cordata extracts can reduce IL-1β levels, and oregano oil can reduce IL-1β and TNF-α levels in broilers [37,38]. In this study, the oregano oil combined with macleaya cordata oral solution reduced serum levels of TNF-α, IL-1β, IL-4, and IL-6, suggesting that the oral solution can inhibit inflammation. A future study should examine in-depth the signaling pathways of oregano oil combined with macleaya cordata oral solution to inhibit inflammation.

The development of small intestinal morphology is directly related to the absorptive capacity of animals. The height of the villus, crypt depth, and the ratio of the villus to the crypt are important indices to evaluate the shape of the small intestine and measure the digestive and absorption function of the intestinal tract. A greater villus height increases the absorption area of the small intestine. The deeper the crypt is, the slower the digestion and absorption function and the larger the villus height/crypt depth ratio is, the stronger the digestion and absorption ability [39,40,41]. Our results showed that the oregano oil combined with macleaya cordata oral solution can significantly improve intestinal morphology and digestive absorption capacity, which is consistent with the results of this study on improving the growth capacity of broilers.

Interestingly, in our study, oregano oil oral solution or macleaya cordata oral solution alone had limited effects on improving growth capacity, serum biochemical indices, immune indices, cytokines, and intestinal morphology of broilers. This phenomenon may be because previous studies have used much higher doses than ours, and previous studies have mainly used additives in feed [19,32]. The efficacy of essential oils may be influenced by various factors such as the content of bioactive ingredients, processing methods, physicochemical properties, plant parts, and interactions with other feed ingredients [42,43]. Further studies are needed to investigate the potential mechanism of oregano oil combined with macleaya cordata oral solution to improve nutrient utilization. However, we added it to drinking water in the form of oral liquid, and some studies showed that the effect of adding it to drinking water was better than that of adding it to feed. Adding drugs into drinking water was more beneficial to the absorption of broilers and reduced the dosage of drugs, thus playing a greater role [29,44].

## 5. Conclusions

In conclusion, this study was the first to study the effect of oregano oil combined with macleaya cordata oral solution on the growth of broilers. The oregano oil combined with macleaya cordata oral solution significantly improved the growth performance of broilers. At the same time, serum biochemical indices, serum antioxidant indices, serum immune indices, serum cytokines, and intestinal morphology were significantly improved by the oregano oil combined with macleaya cordata oral solution. In summary, the mixed solution of oregano oil and macleaya cordata has great potential as an alternative to AGPs for broilers, which can reduce costs and improve benefits. This study provides basic data and technical support for further research.

## Figures and Tables

**Table 1:** Composition and nutrient levels of the basal diet (air-dry basis).

Ingredients	%	Nutrient Levels	% ^3^
Corn	60.00	Metabolic energy, MJ/kg	13.25
Soybean meal	28.40	Crude protein	21.00
Cottonseed meal	7.15	Crude fiber	7.00
Limestone	1.80	Crude ash	8.00
CaHPO4	1.60	Ca	1.00
NaCl	0.35	Total phosphorus	0.45
Lysine	0.35	Lysine	0.95
Vitamin premix ^1^	0.20		
Trace element premix ^2^	0.15		
Total	100.00		

^1^ The premix provided the following per kg of the diet: Fe 60 mg, Cu 10 mg, Zn 45 mg, Mn 60 mg; ^2^ The premix provided the following per kg of the diet: VA 5000 IU, VD3 1000 IU, VE 10 IU, VB2 3.6 mg, VB12 0.01 mg; ^3^ Nutrient levels were all calculated values.

## Data Availability

The study’s original contributions are included in the article; further inquiries can be directed to the corresponding authors.

## Supplementary Materials

The following supporting information can be downloaded at: https://www.mdpi.com/article/10.3390/ani12182480/s1, Table S1: Oregano oil combined with macleaya cordata oral solution improves the growth performance and immune response of broilers
